# Soil Fauna Accelerate Litter Decomposition of *Cry1Ac* Transgenic Maize Independently of Microbial α-Diversity: A Multi-Year, Multi-Region Field Study

**DOI:** 10.3390/ani16193064

**Published:** 2026-09-29

**Authors:** Baifeng Wang, Shijie Li, Zhilei Jiang, Fengci Wu, Junqi Yin, Xinyuan Song

**Affiliations:** 1 Institute of Agricultural Biotechnology, Jilin Academy of Agricultural Sciences (Northeast Agricultural Research Center of China), Changchun 130033, Chinajiang@jaas.com.cn (Z.J.);; 2 Jilin Provincial Crop Transgenic Science and Technology Innovation Center, Changchun 130033, China

**Keywords:** soil fauna, microorganisms, Bt maize, leaf litter decomposition

## Abstract

The return of Bt transgenic maize litter to farmland may affect soil fauna and litter decomposition. From 2015 to 2018, we carried out a multi-year, multi-region litterbag experiment in three major maize-growing regions of China using *Cry1Ac* transgenic maize Bt-799 and its non-transgenic hybrid. We used litterbags with mesh sizes of 5, 2, and 0.02 mm to control soil fauna access. Soil fauna communities and litter decomposition rates were examined in all regions, and bacterial and fungal communities were additionally examined in the third year at Gongzhuling. The results showed that the introduction of *Bt* gene had no significant effect on soil fauna community composition, microbial α-diversity, or litter decomposition. Decomposition rates were significantly higher in large- and medium-mesh litterbags than in small-mesh litterbags across all regions and years, demonstrating that soil fauna substantially accelerated litter decomposition. Soil fauna abundance was significantly positively correlated with decomposition rate (r = 0.847, *p* < 0.001), whereas maize variety and microbial α-diversity indices showed no significant correlation. These findings indicate that soil fauna promote litter decomposition of *Cry1Ac* transgenic maize, with no detectable association with microbial α-diversity, and that the *Bt* gene has no detectable effect on the measured soil biological processes.

## Simple Summary

Genetically modified insect-resistant maize is now cultivated on a large scale globally, and its post-harvest leaf residues remain in farmland soils. It is critical to clarify whether these residues affect soil-dwelling animals and microorganisms, as well as how soil organisms drive the breakdown of dead plant material. We carried out multi-year field experiments across three major maize-growing regions of China. We compared insect-resistant transgenic maize with its non-transgenic counterpart and deployed litterbags with different mesh sizes to regulate access by soil animals. Our results indicate that the transgenic modification produced no measurable shifts in soil-animal communities or microbial diversity and did not change how quickly maize leaf litter decomposed. In contrast, soil animals greatly accelerated plant-residue breakdown: litter decomposed faster when soil animals could enter the litterbags. Soil-animal abundance was closely linked to decomposition speed, whereas such a relationship was not observed for microbial diversity. These findings suggest that soil animals promote litter decomposition independent of measurable shifts in microbial diversity. This study provides empirical field evidence that insect-resistant transgenic maize residues exert no detectable adverse effects on soil biota under our test conditions, and highlights the central role of soil animals in nutrient cycling within agricultural ecosystems.

## 1. Introduction

Bt transgenic maize utilizes genes derived from *Bacillus thuringiensis* to control harmful insect pests such as maize borers. Cultivating Bt maize can significantly reduce the application of chemical insecticides and lower labor input [1,2]. The environmental impacts of Bt crops have long been a core focus of agroecological risk assessment [3,4]. Most previous studies have concentrated on aboveground ecosystems and surface processes [5,6,7].

However, with the expansion of Bt crop cultivation and the increasingly widespread adoption of straw return practices, Bt proteins can enter soil ecosystems via plant rhizospheres and decomposing crop residues [8,9]. It remains unclear whether Bt proteins that enter the soil can be degraded rapidly. Existing research shows that the degradation of Bt proteins in soil is associated with crop varieties, exogenous gene types, planting patterns, and environmental conditions [10,11,12]. Thus, it is necessary to clarify whether undegraded Bt proteins have adverse effects on the soil environment.

Most previous studies on the below-ground effects of Bt crops were only conducted in a single region, spanning one or a few growing seasons and focusing mainly on the crop growth period [13,14,15,16]. However, the soil environment plays a critical role in nutrient cycling and energy flow within ecosystems [17,18]. As important components of soil ecosystems [19,20], soil fauna are widely used as bioindicators in soil ecological research [21,22].

Several studies have already examined the decomposition of Bt maize residues and their effects on soil biota, including multi-year field assessments of soil microarthropod communities [13], investigations of Bt-maize residue decomposition and the associated soil meso- and macrofauna [23], field studies on Bt maize leaf litter and soil Collembola in Northeast China [24], and comprehensive reviews of the fate and effects of Bt crops in soil ecosystems [25]. Nevertheless, to the best of our knowledge, no study has simultaneously quantified Bt maize litter decomposition, soil fauna communities, and microbial community diversity across multiple years and multiple major maize-growing regions. Therefore, long-term, multi-region studies that integrate these measurements are essential to provide a stronger basis for evaluating the ecological safety of Bt crops.

Bt protein exerts its toxicity through specific receptor-mediated binding in the midgut of susceptible lepidopteran larvae [26]; because the corresponding receptors are absent in non-target soil organisms, direct toxicity to soil fauna would not be expected a priori. Nevertheless, some laboratory bioassays have reported adverse effects of Bt proteins on non-target soil biota [27,28], whereas most field studies have failed to detect significant effects [13,29]. These conflicting laboratory and field observations may reflect differences in exposure dose, environmental complexity, and the in situ degradation dynamics of the protein, and they leave genuine uncertainty about the ecological effects of Bt residues under realistic field conditions. An alternative possibility must also be considered: rather than being inert, Bt protein entering the soil could suppress soil fauna activity and thereby indirectly weaken fauna-driven litter decomposition.

Soil fauna can directly influence litter decomposition through feeding, physically breaking down and digesting litter [30,31]. They can also indirectly affect decomposition by altering microbial communities [32], while microorganisms contribute to litter decomposition [33,34,35]. However, it remains unclear whether changes in soil fauna affect Bt litter decomposition and whether their promoting effects are mediated by microorganisms. Mechanistically, litter decomposition is driven jointly by soil fauna and microorganisms, which can act through direct (faunal feeding and physical fragmentation) and indirect (microbe-mediated) pathways. Distinguishing these pathways requires that litter decomposition, soil fauna communities, and microbial diversity be quantified simultaneously within the same experimental system; measuring only one component would leave the causal pathway ambiguous. Testing these competing expectations, rather than treating the hypotheses as post hoc expectations, requires the joint assessment of all three compartments.

Accordingly, we conducted a three-year litterbag experiment across three major maize-growing regions in China, using *Cry1Ac* transgenic maize Bt-799 and its non-transgenic hybrid Zheng 58 as research materials. This study aimed to explore the responses of soil fauna, litter decomposition processes, microbial dynamics, and the mechanisms through which soil fauna regulate litter decomposition. We proposed the following hypotheses: (1) The introduction of the *Bt* gene does not alter soil faunal community composition, microbial diversity, or litter decomposition rates across major maize-growing regions; (2) soil fauna significantly accelerate litter decomposition in both Bt and non-Bt maize systems, regardless of maize variety; and (3) soil fauna promote litter decomposition independent of changes in microbial community diversity.

## 2. Materials and Methods

### 2.1. Experimental Design

This study was divided into two parts.

Part 1 was a two-year experiment carried out simultaneously in three major maize-growing regions of China: (1) Gongzhuling, Jilin Province (124°49′ E, 43°32′ N; mean annual temperature: 5.6 °C; Northeast China Spring Maize Region); (2) Shangzhuang, Beijing (116°10′ E, 40°08′ N; mean annual temperature: 12.5 °C; North China Spring Maize Region); and (3) Xinxiang, Henan Province (113°54′ E, 35°18′ N; mean annual temperature: 14.9 °C; Huanghuaihai Summer Maize Region). The basic physicochemical properties of the soil in these regions differ significantly. Two factors were set in the experimental design: maize type (Bt transgenic maize and its corresponding non-transgenic hybrid), and litterbag mesh size (5 mm, 2 mm, and 0.02 mm), which are designed to control the access of different groups of soil fauna to the litter. The 5 mm mesh allowed access for almost all soil fauna, the 2 mm mesh only allowed access for small and medium-sized fauna, and the 0.02 mm mesh excluded nearly all soil fauna [36]. This part of the experiment focused on two core questions: (1) whether Bt maize residues affect soil faunal communities; and (2) how the introduction of the *Bt* gene and the presence of soil fauna influence litter decomposition.

Part 2 was a one-year experiment that was only carried out in Gongzhuling, Jilin Province, with the same experimental treatments as Part 1. In addition to addressing the research objectives carried over from Part 1, this section focused on two additional questions: (1) whether Bt maize residues affect soil microbial communities; and (2) whether the effect of soil fauna on litter decomposition is associated with microbial diversity.

### 2.2. Experimental Materials

The maize materials used in both parts of this experiment were collected in 2015 from the experimental fields of the Gongzhuling Branch of Jilin Academy of Agricultural Sciences. The materials included *Cry1Ac* transgenic maize Bt-799 and its corresponding non-transgenic hybrid Zheng 58. On 8 October 2015, mature maize leaves were collected and dried in an oven at 40 °C for 72 h. The dried leaves were either used immediately or stored at −20 °C for later use and were re-dried at 40 °C for 24 h before the experiment.

### 2.3. Experimental Layout

#### 2.3.1. Part 1

The dried maize leaves were cut into 8 cm segments. For the 2015 and 2016 experiments, 4.0 g of processed leaf material was placed into each 15 cm × 20 cm litterbag.

At each study region, three 5 m × 6 m replicate blocks were set up each year following a randomized block design, with a 2 m buffer zone between adjacent blocks. All experimental sites had been conventionally managed as maize fields prior to the experiment, and no crops were planted on the sites during the experimental period; manual weeding was performed every 10 days throughout the study.

Litterbags were buried between 15–19 November 2015 (15 bags per treatment per block) and 10–15 November 2016 (12 bags per treatment per block). Burial positions were randomly selected within each block, with a minimum distance of 2 m between positions. One litterbag per treatment was placed at each position, and all litterbags were buried vertically at a depth of 5–10 cm and marked with plastic tags.

#### 2.3.2. Part 2

In 2017, litter preparation followed the same procedure as in previous years, except that 6 g of litter material (including 2 g reserved for microbial analysis) was placed in each 18 cm × 25 cm litterbag. The larger bag size and increased litter mass (6 g per bag) were adopted in Part 2 to ensure we obtained sufficient litter material for parallel decomposition measurements and the reserved microbial analyses (2 g per bag), while the litter loading per unit bag area remained comparable to that in Part 1. On 13 November 2017, litterbags were buried only at the Gongzhuling site, with nine bags per treatment per block.

### 2.4. Sample Collection and Processing

#### 2.4.1. Part 1

From 1 April to 1 August 2016, samples were collected at 30-day intervals, (five sampling times in total. At each sampling time, three litterbags per treatment were collected from each block. Soil fauna were separated from the litter residues in large- and medium-mesh litterbags using the Berlese–Tullgren dry-funnel method. Extraction was conducted for 7 days at room temperature (20–30 °C). After extraction, the separated fauna were preserved in 75% ethanol, identified to the broad taxon level under an SZ51 stereomicroscope (Olympus Corporation, Tokyo, Japan), and counted to calculate individual abundance.

Subsequently, all treated litter residues were cleaned, dried at 40 °C for 72 h, and weighed individually. Next, residues from large-mesh litterbags were ground, and samples from the same block were pooled. Bt protein content was then measured using a Cry1Ab/Ac ELISA kit (Agdia, Inc., Elkhart, IN, USA).

Between 15 April and 15 July 2017, sampling was carried out every 30 days (four sampling events in total) following the same procedures used in 2016.

#### 2.4.2. Part 2

From 15 April to 15 June 2018, samples were collected at Gongzhuling at 30-day intervals, with three sampling events in total. Two-thirds of the collected samples were processed following the procedures described in Part 1. The remaining one-third of the litter residue from each bag was immediately frozen in dry ice and stored at −80 °C for subsequent microbial analysis. Before analysis, the litter residue was removed from the freezer and left to stand for 10 min. After thawing, each sample of litter residue was mixed with 15 mL of PBS buffer. The mixture was shaken at 200 rpm for 30 min, sonicated for 5 min, then shaken again for an additional 30 min. Following centrifugation at 10,000× *g* for 5 min, the supernatant was discarded, and the pellet along with the residual liquid (approximately 1 mL) at the bottom of the tube was collected for total DNA extraction, and subsequent high-throughput sequencing of the bacterial 16S rRNA gene (V3–V4 region) and the fungal ITS1 region was performed.

### 2.5. DNA Extraction, PCR Amplification, and Sequencing

Total genomic DNA was extracted from the pellet collected in Section 2.4.2 using the VAMNE (Vazyme Biotech Co., Ltd., Nanjing, China; catalog no. DM401-C3-P2) according to the manufacturer’s instructions. DNA concentration was measured using the dsDNA HS assay (1× dsDNA HS Working Solution; Yeasen Biotechnology (Shanghai) Co., Ltd., Shanghai, China) on a Synergy HTX microplate reader (Gene Company Limited, Hong Kong, China). The V3–V4 hypervariable region of the bacterial 16S rRNA gene and the fungal ITS1 region were amplified with the primer pairs 338F (5′-ACTCCTACGGGAGGCAGCA-3′)/806R (5′-GGACTACHVGGGTWTCTAAT-3′) and ITS1F (5′-CTTGGTCATTTAGAGGAAGTAA-3′)/ITS2 (5′-GCTGCGTTCTTCATCGATGC-3′), respectively. PCR was performed in a 10 μL reaction system containing 2.5–4 ng of genomic DNA, 0.3 μL of each primer (10 μM), 5 μL of 2× KOD FX Neo buffer, 2 μL of dNTPs (2 mM each), and 0.2 μL of KOD FX Neo DNA polymerase (1.0 U; Toyobo Co., Ltd., Osaka, Japan), using the following cycling conditions: initial denaturation at 95 °C for 5 min; 25 cycles of denaturation at 95 °C for 30 s, annealing at 50 °C for 30 s, and extension at 72 °C for 40 s; and a final extension at 72 °C for 7 min. The target-region PCR products were purified with VAHTS DNA Clean Beads (Vazyme Biotech Co., Ltd.), and dual-indexed sequencing adapters were added by an indexing PCR. The final library was quantified using the KAPA Library Quantification Kit (KAPA Biosystems, Wilmington, MA, USA) on a LightCycler 480 II real-time PCR system (RocheDiagnostics GmbH, Mannheim, Germany), and paired-end (PE) sequencing was performed on an Illumina NovaSeq 6000 platform (Illumina Inc., San Diego, CA, USA) with a small-fragment library sequencing strategy.

Raw paired-end reads were quality-filtered with Trimmomatic v0.33 using a 50 bp sliding window, and reads were truncated when the average quality within the window fell below 20; primer sequences were then identified and removed using cutadapt v1.9.1 (maximum mismatch rate 20%, minimum coverage 80%). Clean reads were assembled into tags by overlapping the paired-end reads with USEARCH v10 (minimum overlap length 10 bp, minimum overlap identity 90%), and chimeric sequences were identified and removed with UCHIME v4.2. The effective sequences were clustered into operational taxonomic units (OTUs) at 97.0% sequence similarity using USEARCH v10, and OTUs accounting for less than 0.005% of the total number of sequences were filtered out.

### 2.6. Data Analysis

Species richness, abundance, Simpson’s diversity index, Shannon–Wiener index, and Pielou’s evenness index for soil fauna were calculated using R software v4.4.1. For bacteria and fungi, alpha diversity indices were calculated using QIIME2 v2023.7.

Statistical analyses were performed using SPSS v23.0. Before formal analysis, we rigorously assessed the normality of variable distributions, linear relationships between variables, and potential outliers. Three-way ANOVA with maize variety, litterbag mesh size, and sampling time as fixed factors was performed separately for each year and region. Data normality and homogeneity of variances were verified using the Shapiro–Wilk test and Levene’s test, respectively, before ANOVA. When significant differences were identified, post hoc multiple comparisons were conducted using the LSD test. Partial correlation analysis was applied to explore the correlations between litter decomposition rate and key biotic variables, including soil fauna abundance and microbial diversity indices, with sampling time set as the control variable. Specifically, the partial correlation coefficients were calculated using the Partial Corr procedure in SPSS (Pearson partial correlation), and two-tailed *p* values were reported; the analysis was based directly on the correlation matrix and was not derived from regression modelling.

## 3. Results

### 3.1. Part 1

#### 3.1.1. Decomposition of Bt Protein in Bt Maize Litter

**Figure 1:**
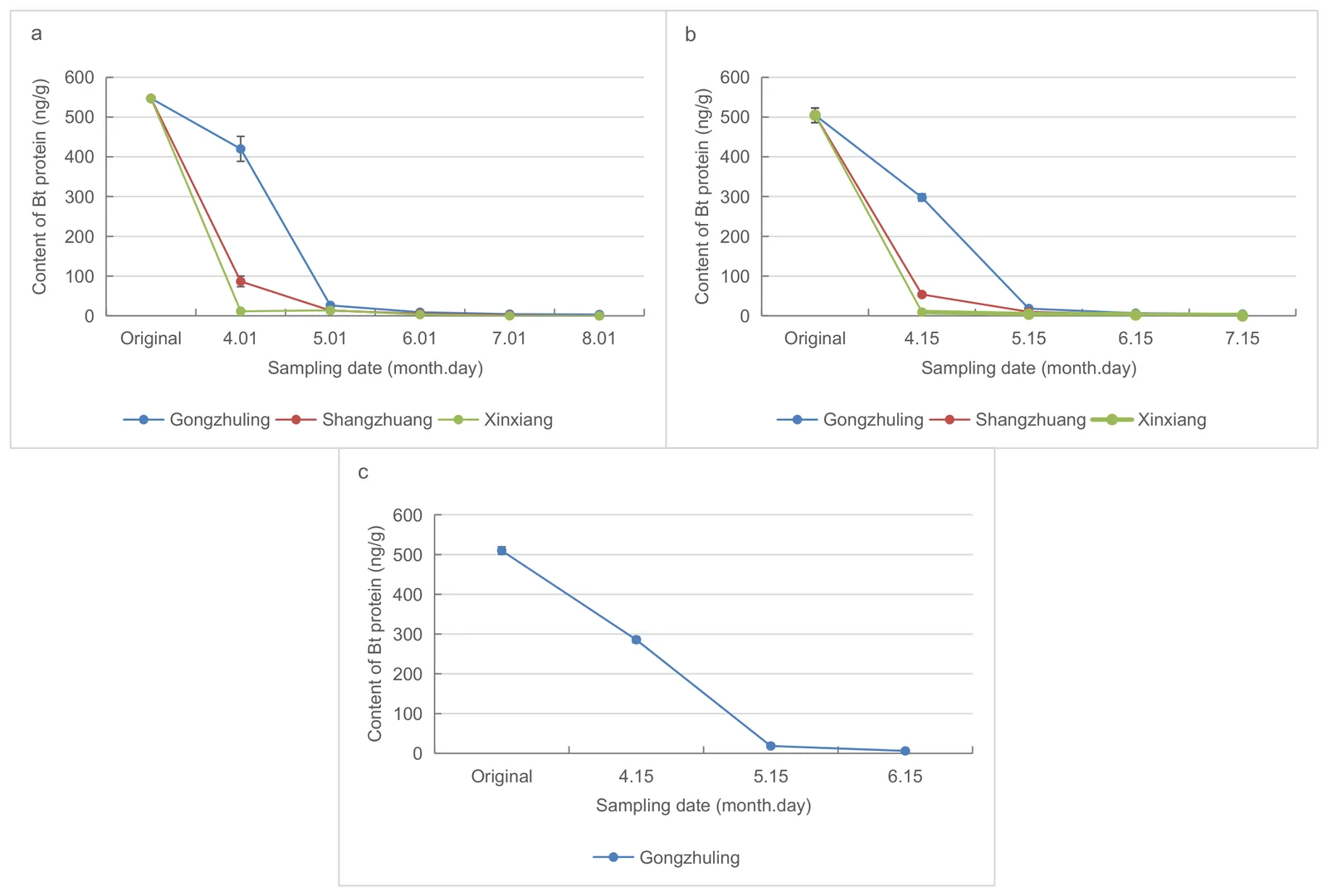
Temporal degradation dynamics of Bt protein residues in large-mesh litterbags across experimental sites, 2016–2018. (**a**) 2016 experiment (Gongzhuling, Shangzhuang, Xinxiang); (**b**) 2017 experiment (Gongzhuling, Shangzhuang, Xinxiang); (**c**) 2018 experiment (only Gongzhuling site). Values are means ± standard errors (SE).

Bt protein content in large-mesh litterbags was measured using a Cry1Ab/Ac ELISA kit. The results showed that the initial Bt protein content in maize leaf litter was (546.37 ± 2.72) ng/g. For samples buried in November 2015, Bt protein content was measured in each sampling period of 2016 across the three experimental regions. After approximately 140 days (1 April 2016), Bt protein was still detectable in all the regions. In Gongzhuling, only a small proportion of Bt protein had degraded by April 1, with a remaining concentration of (420.93 ± 31.26) ng/g, accounting for 77.04% of the initial content. In contrast, most Bt protein had already degraded in Shangzhuang and Xinxiang, with remaining concentrations of (84.79 ± 13.52) ng/g and (11.42 ± 1.39) ng/g, corresponding to 15.53% and 2.09% of the initial content, respectively. After that, Bt protein degraded rapidly in Gongzhuling, dropping to (25.78 ± 4.55) ng/g by 1 May. Small amounts of Bt protein remained detectable in Gongzhuling until 1 August. In Shangzhuang and Xinxiang, only trace amounts remained by May and June, and almost no Bt protein was detected in subsequent sampling periods (Figure 1a).

For samples buried in November 2016, the temporal dynamics of Bt protein in 2017 followed similar patterns to the 2015 burial experiment. By 15 April 2017, the remaining Bt protein content was (297.70 ± 8.98) ng/g in Gongzhuling, while considerably lower levels were detected in Shangzhuang (53.55 ± 4.56 ng/g) and Xinxiang (9.54 ± 0.79 ng/g). Bt protein concentrations dropped to very low levels between 15 May and 15 June. By 15 July, only a small amount of Bt protein was still detectable in Gongzhuling (4.09 ± 0.32 ng/g), while it was almost undetectable in both Shangzhuang and Xinxiang (Figure 1b).

#### 3.1.2. Effects of *Bt* Gene Introduction, Litterbag Mesh Size, and Sampling Time on Soil Fauna

**Figure 2:**
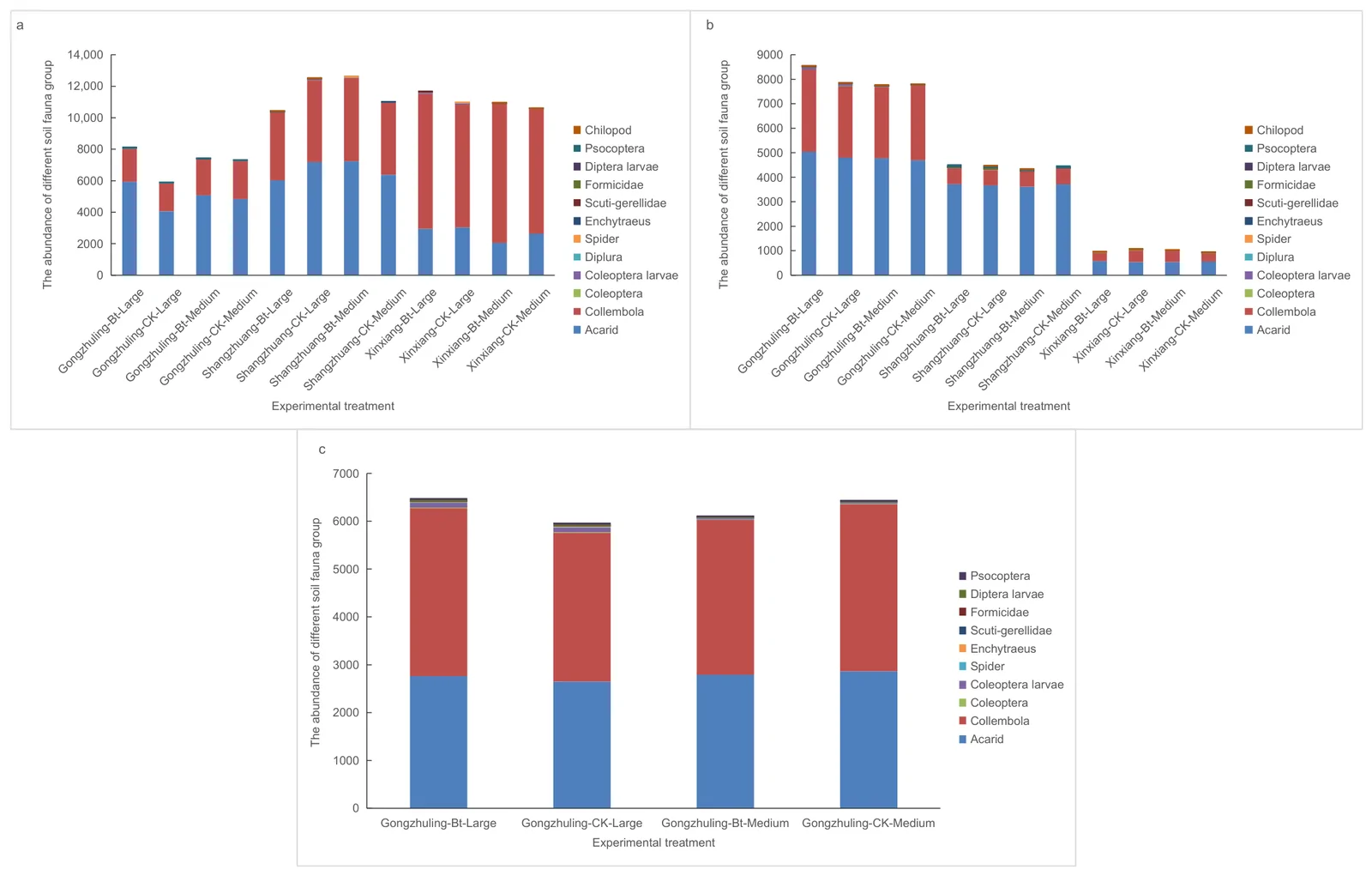
Total abundance and taxonomic composition of soil faunal communities under maize variety and litterbag mesh size treatments across sites and years. Bt = *Cry1Ac* transgenic maize Bt-799; CK = non-transgenic maize Zheng 58; Large, Medium, Small represent three mesh sizes of litterbags: Large = 5 mm, Medium = 2 mm, Small = 0.02 mm. (**a**) Sampling in 2016 across three experimental sites; (**b**) Sampling in 2017 across three experimental sites; (**c**) Sampling in 2018 at Gongzhuling only. Values are means ± SE.

In total, 486 samples were collected across the three regions through nine field surveys conducted in 2016 and 2017. From these samples, we identified 85,051 individual soil fauna belonging to 12 taxonomic groups. Acarida and Collembola were the dominant groups, accounting for 99.34% of the total individual abundance. We aggregated soil fauna data by sampling year, study site and treatment, and the abundances of different taxa are shown in Figure 2.

The effects of maize variety, mesh size, and sampling time on five soil fauna community indices (species richness, abundance, Simpson’s diversity index, Shannon–Wiener index, and Pielou’s evenness index) were evaluated using three-way ANOVA across three study regions (Gongzhuling, Shangzhuang, and Xinxiang) from 2016 to 2017 (Table 1).

Across all years and study regions, sampling time consistently exerted a highly significant effect (*p* < 0.01) on almost all measured soil fauna indices. In 2016 specifically, sampling time significantly affected species richness, abundance, Simpson’s diversity index, Shannon–Wiener index, and Pielou’s evenness index at all three sites (all *p* < 0.01). This pattern was repeated in 2017: sampling time significantly affected almost all indices across the three sites of Gongzhuling, Shangzhuang, and Xinxiang, with most *p* values less than 0.01. The only exceptions were Pielou’s evenness index in 2017 and species richness index in 2018 at Gongzhuling, where the effects of sampling time were not significant.

In contrast, neither maize variety nor mesh size had a significant effect on any soil fauna index in any year or region (all *p* > 0.05). For maize variety, *p*-values ranged from 0.21 to 1.00 across all indices, which indicates that maize variety has no consistent influence on soil fauna communities. Similarly, mesh size also had no significant impact, with all *p*-values greater than 0.10 across all sites and years.

#### 3.1.3. Effects of *Bt* Gene Introduction and Soil Fauna on Litter Decomposition

**Figure 3:**
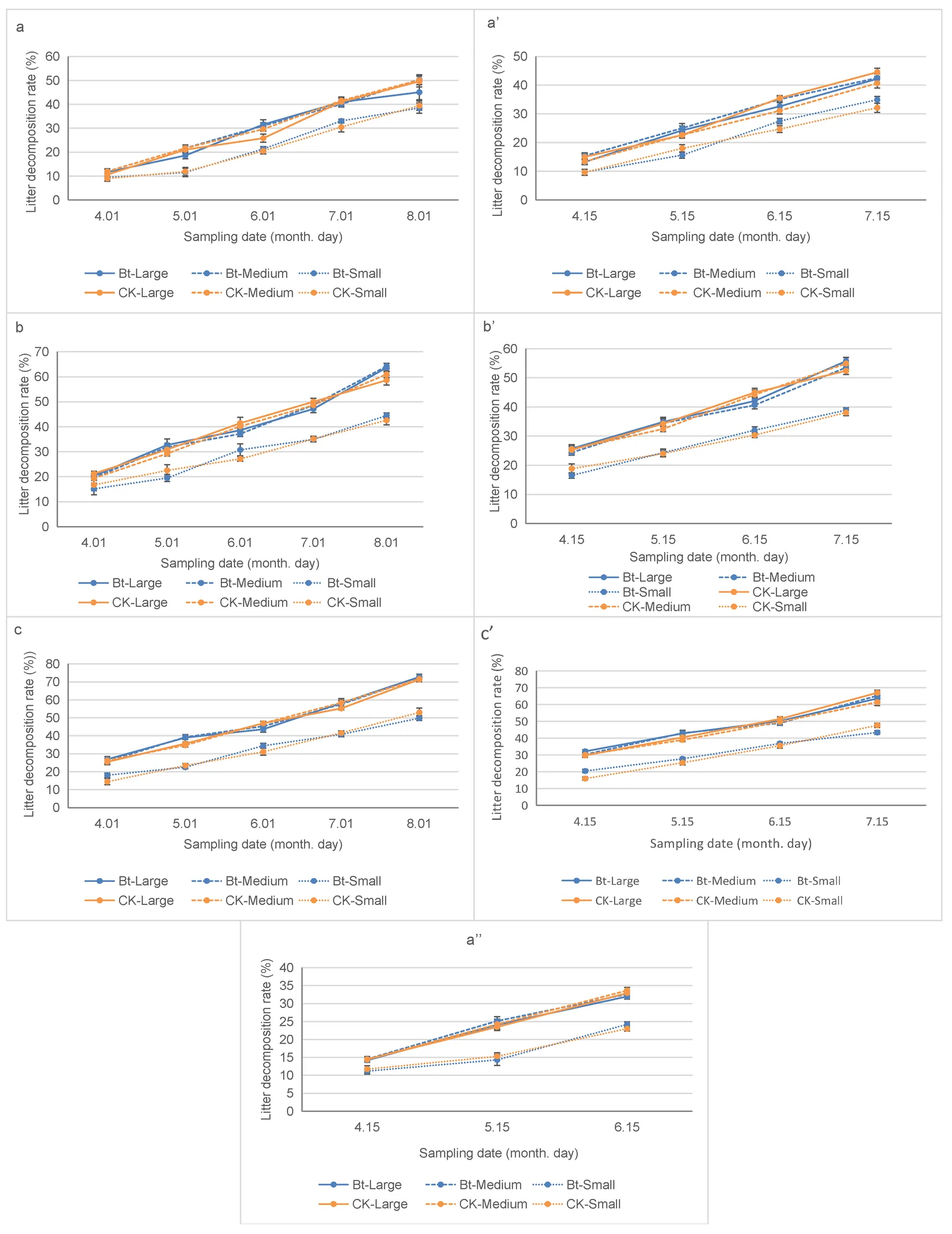
Temporal leaf litter decomposition rates of transgenic and non-transgenic maize under three litterbag mesh sizes across experimental regions, 2016–2018. Bt = *Cry1Ac* transgenic maize Bt-799; CK = non-transgenic maize Zheng 58; Large, Medium, Small represent litterbags with different mesh apertures. (**a**) Data from 2016 in Gongzhuling; (**b**) Data from 2016 in Shangzhuang; (**c**) Data from 2016 in Xinxiang; (**a’**) Data from 2017 in Gongzhuling; (**b’**) Data from 2017 in Shangzhuang; (**c’**) Data from 2017 in Xinxiang; (**a”**) Data from 2018 in Gongzhuling. Values are means ± SE.

Litter decomposition rates were calculated across different regions and years, and the results are shown in Figure 3. Three-way ANOVA was performed to evaluate the effects of maize variety, mesh size and sampling time on litter decomposition rates. The results indicated that sampling time and mesh size significantly affected litter decomposition rates across all study years in all three regions (all *p* < 0.01), while maize variety had no significant effect (all *p* > 0.05) (Table 2). Post hoc LSD tests were conducted to further clarify differences in decomposition rates among different mesh sizes. The results confirmed that decomposition rates in small-mesh litterbags were consistently and significantly lower than those in large- or medium-mesh litterbags (*p* < 0.01), while no significant difference was found between large- and medium-mesh sizes (Table 3).

### 3.2. Part 2

#### 3.2.1. Bt Protein Decomposition, Soil Fauna, and Litter Decomposition

The results presented in Part 2 were consistent with those in Part 1. In Gongzhuling, the Bt protein content was (285.83 ± 9.18) ng/g on 15 April 2018, which accounted for 52.30% of the initial content, and it decreased rapidly after this time point. By 15 May, the Bt protein concentration had dropped to (18.60 ± 1.39) ng/g, and by 15 June, only a trace amount (6.20 ± 0.50 ng/g) remained (Figure 1c).

No significant differences in soil fauna diversity indices were detected across all treatments (*p* > 0.05, Table 1). Similarly, maize variety had no significant effect on litter decomposition, while mesh size and sampling time both showed significant effects (*p* < 0.01, Table 2).

#### 3.2.2. Effects of *Bt* Gene and Litterbag Mesh Size on Microbial Communities

**Figure 4:**
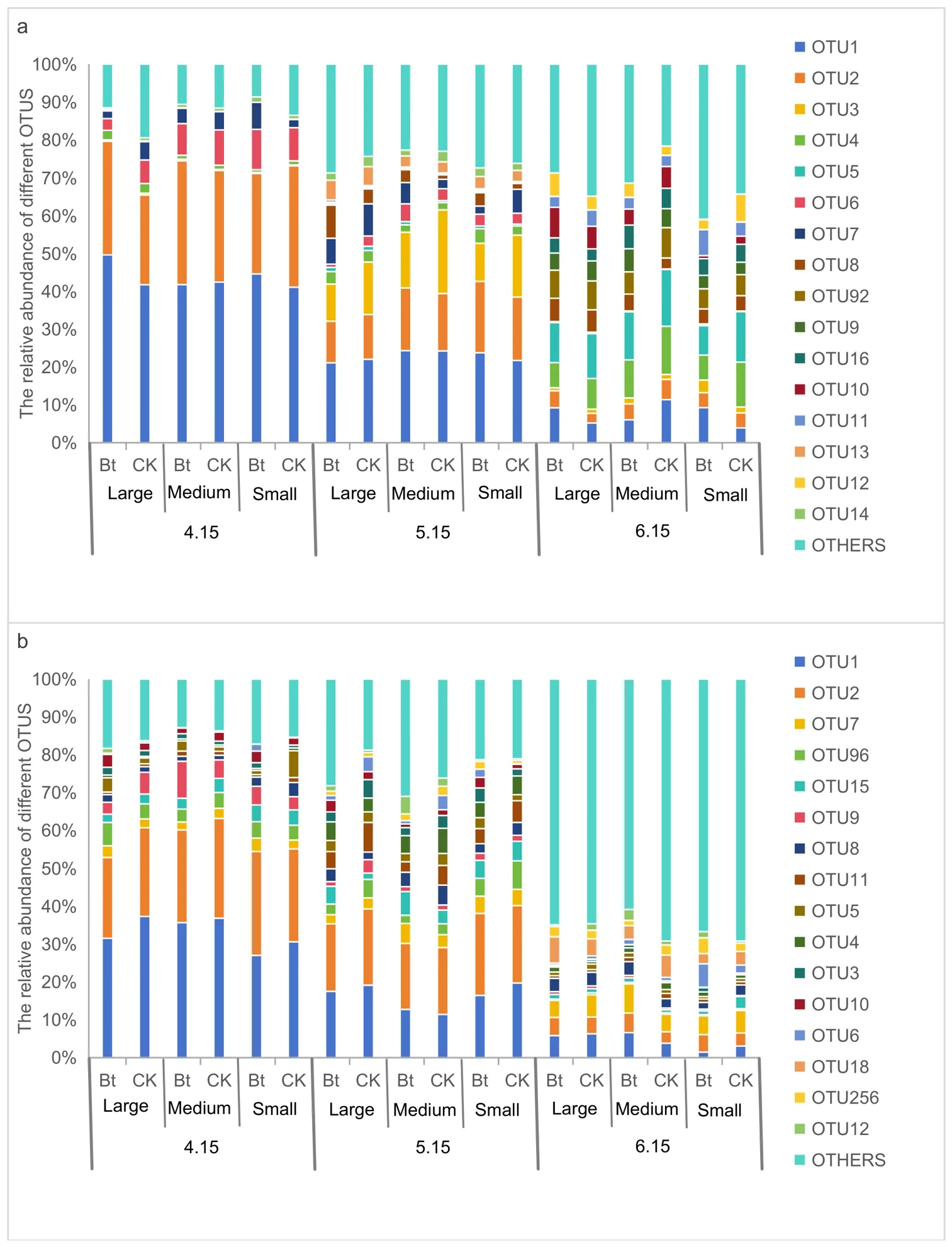
Relative abundance of dominant fungal and bacterial OTUs under different maize varieties, litterbag mesh sizes and sampling dates in 2018 in Gongzhuling. (**a**) Fungal OTUs (ITS1 sequencing); (**b**) Bacterial OTUs (16S rRNA V3 + V4 sequencing). OTU = operational taxonomic unit; “OTHERS” indicates the pooled low-abundance OTUs with negligible individual proportions.

In 2018, 54 samples were collected across three sampling periods. Fungal and bacterial sequences were clustered into OTUs at 97% similarity using USEARCH. The 16 most abundant OTUs were analyzed individually, while all remaining OTUs were pooled into the group “Others”. After aggregating OTUs from the same treatment, the relative abundances of fungal OTUs are presented in Figure 4a, and those of bacterial OTUs are presented in Figure 4b.

Three-way ANOVA was performed to examine the effects of maize variety, mesh size, and sampling time on the abundance of dominant fungal (Table S1) and bacterial (Table S2) OTUs. The results showed that sampling time significantly affected the abundance of all dominant fungal OTUs (*p* < 0.01) and nearly all bacterial OTUs (*p* < 0.01 for most). Maize variety and mesh size only had minor, OTU-specific impacts, with no widespread effects on community composition.

**Figure 5:**
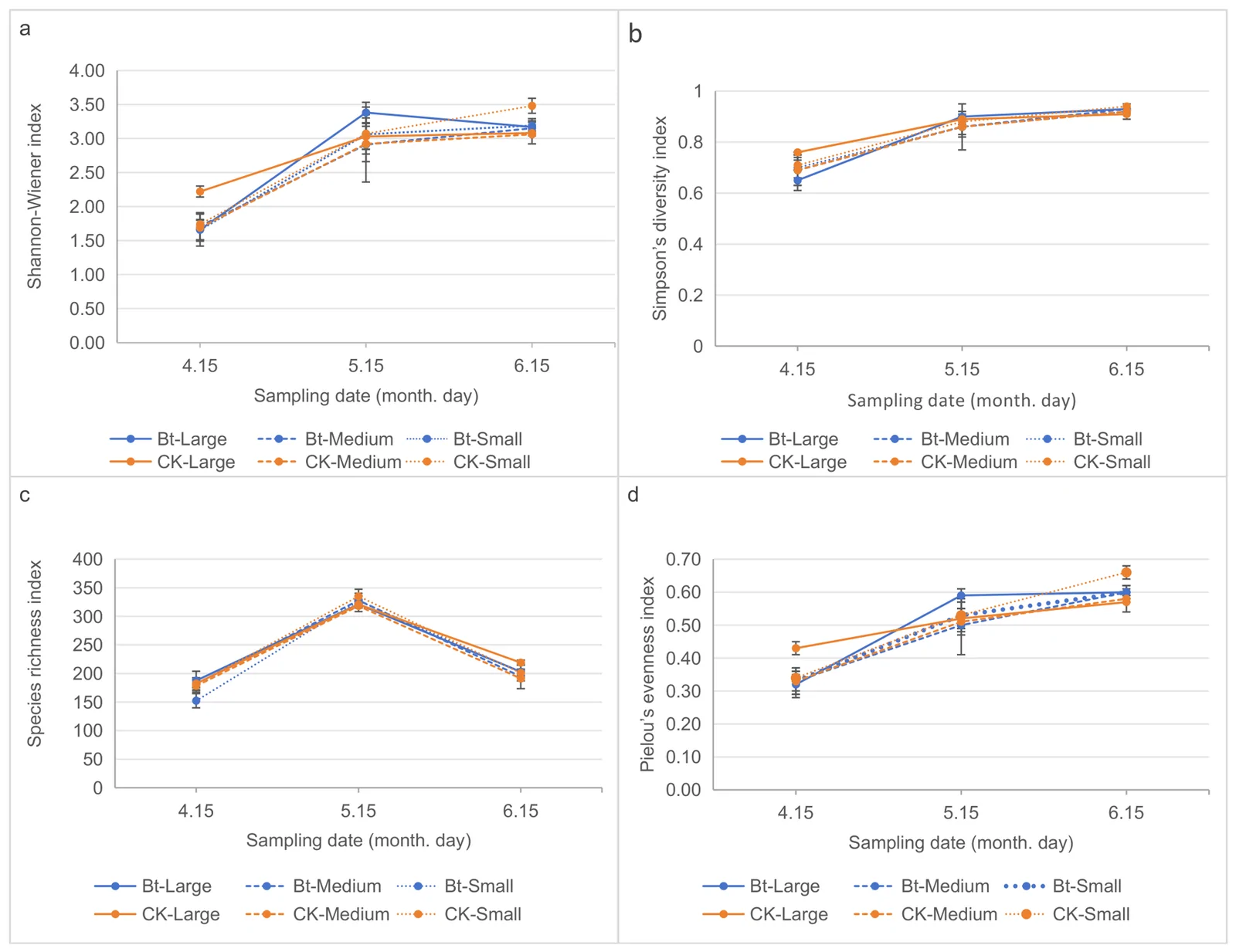
Alpha diversity indices of fungal communities across maize varieties, litterbag mesh sizes and sequential sampling dates in 2018 in Gongzhuling. (**a**) Shannon–Wiener diversity; (**b**) Simpson’s diversity; (**c**) species richness; (**d**) Pielou’s evenness. Values are means ± SE.

**Figure 6:**
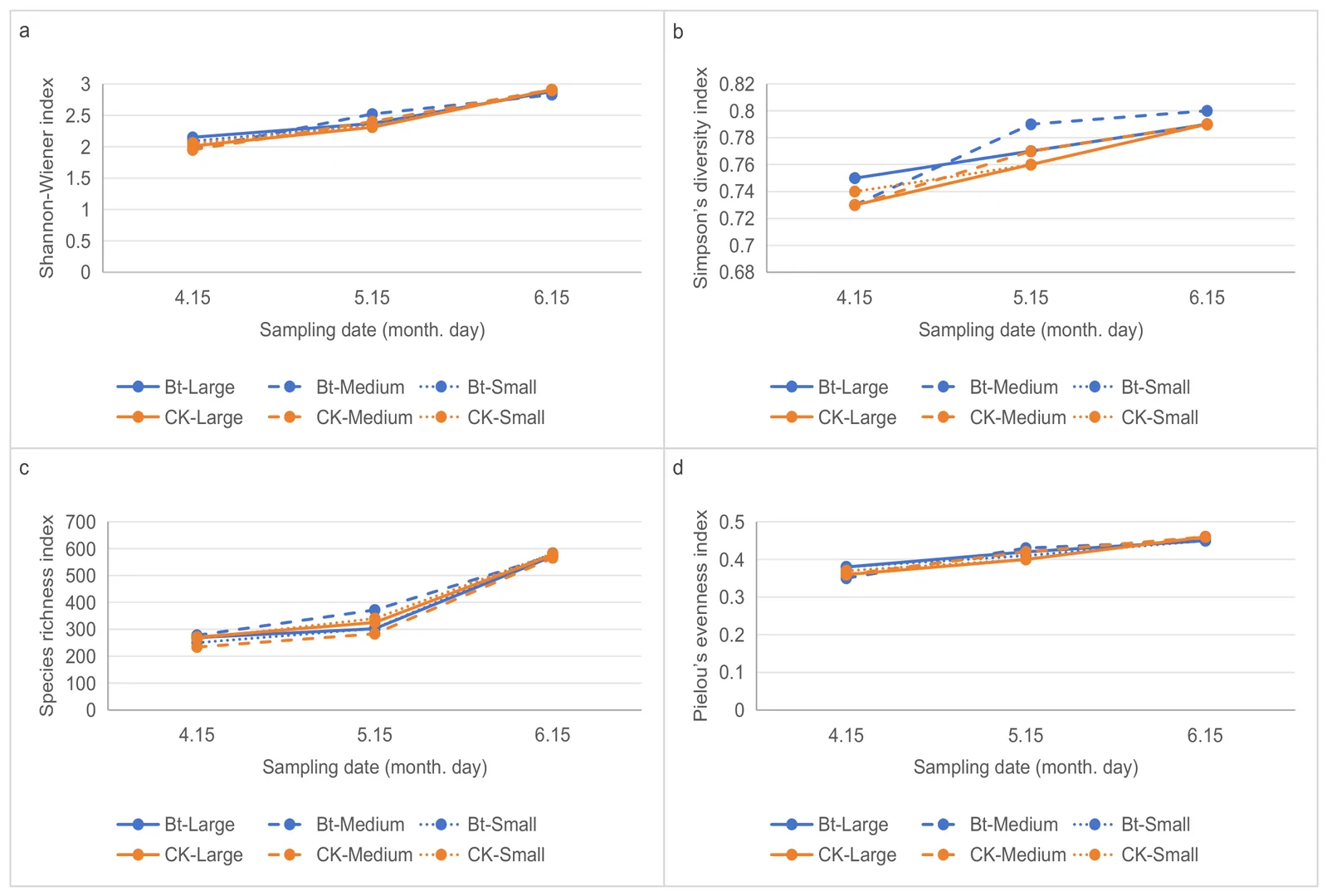
Alpha diversity indices of bacterial communities across maize varieties, litterbag mesh sizes and sequential sampling dates in 2018 in Gongzhuling. (**a**) Shannon–Wiener diversity; (**b**) Simpson’s diversity; (**c**) species richness; (**d**) Pielou’s evenness. Values are means ± SE.

Temporal dynamics of fungal and bacterial community diversity were characterized across three sampling dates (15 April, 15 May, 15 June) for two maize varieties (Bt-799 and Zheng 58) and three mesh sizes (large, medium, and small), using Shannon–Wiener index, Simpson’s diversity index, species richness and Pielou’s evenness index. The fungal results are presented in Figure 5, and the bacterial results are presented in Figure 6. Fungal data showed that all diversity indices of fungal communities peaked in mid-May, followed by a slight decline, whereas bacterial data showed that all four diversity indices increased steadily until June. Neither maize variety nor mesh size significantly changed these overall temporal patterns for either microbial group.

Three-way ANOVA was performed to analyze the effects of maize variety, mesh size, and sampling time on the diversity indices of fungal and bacterial communities (Table 4). The results showed that sampling time significantly affected microbial diversity (*p* < 0.05), while maize variety and mesh size had only minimal effects.

#### 3.2.3. Correlation Analysis

We analyzed the correlation between litter decomposition rate and multiple biotic variables (Table 5) using the 54 samples collected at Gongzhuling in 2018. Partial correlation analysis showed that only soil fauna abundance had a strong, statistically significant positive correlation with litter decomposition rate (r = 0.847, *p* < 0.001), meaning higher soil fauna abundance is associated with accelerated litter decomposition. No significant correlations were detected between litter decomposition rate and maize variety (r = 0.015, *p* = 0.92), nor between litter decomposition rate and any of the fungal or bacterial community metrics (all *p* > 0.05).

## 4. Discussion

The litterbag incubation method is a widely used and reliable approach for investigating litter decomposition processes and belowground ecological interactions in agroecosystems [37,38]. Combining this method with soil fauna surveys and microbial high-throughput sequencing, this study conducted a three-year multi-region field experiment to systematically examine three key aspects: the degradation dynamics of Bt protein in litter of *Cry1Ac* transgenic maize Bt-799, the responses of soil fauna and microbial communities to transgenic litter, and the core mechanisms driving litter decomposition. The results clarified three main conclusions: the Bt protein in the litters will remain in the field for a certain period of time, the negligible impact of the *Bt* gene on soil biota, and the dominant role of soil fauna in promoting litter decomposition independent of changes in microbial community diversity.

In this study, we found that the initial Bt protein content of the tested maize litter was approximately 546 ng/g. Across all study regions and every experimental year, Bt protein remained detectable in the early stage of litter decomposition and declined to low levels by mid-summer (Figure 1). The fact that Bt protein remains detectable throughout the early decomposition stage ensures that soil organisms are fully exposed to the exogenous protein, providing a reliable foundation for ecological safety assessment of transgenic maize.

Three-way ANOVA showed that maize variety exerted no significant effect on any soil faunal community metric in any year or region (all *p* > 0.05; Table 1), and mesh size, which modulates faunal access, likewise did not alter faunal community structure; sampling time was the dominant driver (Table 1). These results demonstrate that Bt protein from Bt-799 litter does not disrupt soil faunal community assembly, which is consistent with findings from most long-term ecotoxicological assessments of Bt crop litter [9,29]. Zurbrügg et al. [39] further reported that the variation in soil faunal communities among conventional maize cultivars was larger than the difference between transgenic and non-transgenic maize lines. Most studies conducted during the maize growing season have also found no significant effects of Bt crops on soil faunal communities [16,40]. Although some studies have detected subtle impacts on sensitive faunal taxa [28,41], our multi-year cross-regional data supports the conclusion that under actual field conditions, Bt maize poses negligible risk to soil faunal communities.

The high dominance of small and medium-sized taxa (Acarid and Collembola, accounting for 99.34% of all individuals; Figure 2) likely explains why mesh size did not produce significant effects, as these groups can easily penetrate both large- and medium-mesh litterbags.

Regarding soil microorganisms, neither maize variety nor mesh size significantly altered the relative abundance of most dominant fungal and bacterial OTUs, nor did it affect microbial diversity indices. In contrast, sampling time was the key factor driving the temporal dynamics of microbial communities. These results are consistent with previous studies, which found that Bt crops have only limited effects on soil microorganisms [42,43,44].

Although a small number of individual OTUs showed nominally significant responses to maize variety or mesh size (e.g., fungal OTU10 to mesh size and bacterial OTU10 to maize variety, *p* < 0.05; Tables S1 and S2), these sporadic OTU-level signals were weak, involved only a small subset of taxa, and were not accompanied by any shifts in community-wide α-diversity. Such scattered responses are consistent with the community-scale resistance of soil microbial assemblages to Bt maize and do not overturn the overall conclusion that the *Bt* gene had no broad effect on soil microbial communities.

Laboratory studies occasionally report adverse effects of Cry proteins on soil biota [27,45], which appear to conflict with the absence of detectable effects in our multi-site field experiment. This discrepancy can be explained by several factors. First, laboratory assays typically expose organisms to high, sustained concentrations of purified proteins that exceed realistic field exposure [45], whereas Bt protein in decomposing litter declined rapidly under field conditions (Figure 1). Second, laboratory microcosms lack the environmental complexity of field soils, where the protein binds to soil particles and is subjected to microbial and enzymatic degradation [25]. Third, field communities may exhibit compensatory dynamics that buffer individual-species responses [46]. These considerations support the view that, under realistic agricultural conditions, Bt maize residues pose negligible risk to soil fauna and microbial communities.

Litter decomposition results mirrored the faunal patterns: maize variety had no significant effect in any region or year, whereas mesh size and sampling time were highly significant, with decomposition in small-mesh bags (excluding nearly all fauna) significantly slower than in large- or medium-mesh bags (Table 2 and Table 3). These results verify that soil fauna, particularly small and medium-sized fauna, can significantly promote the decomposition of maize litter, and that this effect is not affected by transgenic traits.

Most studies have shown that soil fauna significantly facilitate litter decomposition [47,48], and the promoting effect of soil fauna on this process is generally attributed to two mechanisms: (1) direct feeding and physical fragmentation of litter [30,31]; and (2) indirect effects mediated by changes in microbial communities [32]. To determine whether the promoting effect observed in this study is mediated by microorganisms, we performed correlation analysis. The litter decomposition rate was significantly positively correlated with soil fauna abundance (r = 0.847, *p* < 0.001), but showed no significant correlation with maize variety or any microbial α-diversity index (all *p* > 0.05; Table 5). These results indicate that soil fauna significantly promoted litter decomposition with no detectable association with microbial community α-diversity, and that the *Bt* gene did not influence decomposition rates.

The neutral effects of soil fauna on microbial diversity observed in this study are consistent with previous reports of balanced stimulatory and inhibitory interactions between these groups [49,50]. Although mesofauna can boost microbial activity through promoting nutrient mineralization [51,52], they can also reduce microbial biomass via grazing [53,54]. In our study system, these opposing effects appear to offset each other, leaving community-level microbial diversity unchanged. Notably, microorganisms are still fundamental to the biochemical processes of litter decomposition [55,56], but high microbial functional redundancy may buffer decomposition processes against shifts in α-diversity [57]. Taken together, litter decomposition in this system appears to be driven primarily by faunal activity, with no detectable mediation through changes in microbial community α-diversity; however, because only α-diversity indices were assessed and β-diversity and microbial functional traits were not measured, functional mediation by microorganisms cannot be fully ruled out.

The strong positive correlation between soil fauna abundance and litter decomposition rate (r = 0.847, *p* < 0.001) was consistent across mesh sizes, regions, and years. The litterbag mesh-size gradient served as a faunal-exclusion design: litter accessible to soil fauna decomposed consistently faster than litter from which fauna were excluded, and the abundance–decomposition association remained highly significant after controlling for sampling time in the partial correlation analysis. We acknowledge that, because mesh size also modified the litter microenvironment (e.g., moisture and aeration) and because the analyses were correlative, factors other than faunal activity, such as litter microclimate, may have contributed to the observed association. Nevertheless, the strength and consistency of the relationship across three regions and three years, the dose-dependent response along the mesh-size gradient, and the well-established mechanisms by which soil fauna fragment litter and stimulate microbial decomposition collectively support the conclusion that soil fauna played a major role in promoting litter decomposition, independently of changes in microbial community diversity.

Several limitations of this study should be acknowledged. First, the microbial analyses were conducted only in the final year (2018) and at a single site (Gongzhuling), and only α-diversity indices were assessed; β-diversity and microbial functional traits were not measured. The restricted spatial and temporal scope of the microbial analyses therefore limits the generalizability of the microbial results across regions and years, and functional mediation of litter decomposition by microorganisms cannot be fully excluded. Second, the inference that soil fauna promoted litter decomposition independently of microbial community diversity is based on correlation analysis rather than on formal mediation testing or direct measurements of microbial activity; future studies combining faunal manipulation with microbial functional assays and structural equation modeling across additional sites are needed to strengthen causal inference. Third, although the litterbag method is widely used, it cannot fully reproduce natural residue–soil contact, and the smallest mesh size may have restricted the activity of microfauna as well as mesofauna. Despite these limitations, the consistent multi-year and multi-region patterns provide robust evidence for the main conclusions of this study.

## 5. Conclusions

Based on a multi-year, multi-region field study, *Cry1Ac* transgenic maize Bt-799 had no significant effects on soil fauna or microbial community diversity, and *Bt* gene insertion did not affect the decomposition of maize leaf litter.

Soil fauna significantly enhanced litter decomposition, but this effect showed no detectable association with microbial relative abundance or diversity (α-diversity indices), indicating that the faunal promotion of litter decomposition was not detectably linked to changes in microbial community α-diversity under the conditions and measurements of this study.

Overall, these findings provide evidence that Bt maize residues have no detectable adverse effects on the soil biota under the tested conditions and highlight the dominant role of soil fauna in regulating litter decomposition.

## Figures and Tables

**Table 1:** Effects of maize variety, mesh size and sampling time on soil fauna community indices (three-way ANOVA, 2016–2018).

Year	Region	Factor	*p* Value				
			Species Richness	Abundance	Simpson’s Diversity	Shannon–Wiener	Pielou’s Evenness
2016	Gongzhuling	Maize variety	0.65	0.24	0.85	0.83	0.90
		Mesh size	0.29	0.71	0.25	0.26	0.55
		Sampling time	<0.001 ***	<0.001 ***	<0.001 ***	<0.001 ***	<0.001 ***
	Shangzhuang	Maize variety	0.53	0.87	0.53	0.65	0.22
		Mesh size	0.26	0.77	0.42	0.40	0.62
		Sampling time	<0.001 ***	<0.001 ***	<0.001 ***	<0.001 ***	<0.001 ***
	Xinxiang	Maize variety	1.00	0.96	0.63	0.63	0.73
		Mesh size	0.32	0.14	0.85	0.87	0.89
		Sampling time	<0.001 ***	<0.001 ***	<0.001 ***	<0.001 ***	<0.001 ***
2017	Gongzhuling	Maize variety	0.62	0.27	0.58	0.86	0.21
		Mesh size	0.20	0.44	0.30	0.70	0.33
		Sampling time	<0.003 **	<0.001 ***	<0.001 ***	<0.001 ***	0.16
	Shangzhuang	Maize variety	0.57	0.83	0.62	0.53	0.71
		Mesh size	0.19	0.66	0.29	0.18	1.00
		Sampling time	<0.001 ***	<0.001 ***	<0.001 ***	<0.001 ***	<0.001 ***
	Xinxiang	Maize variety	0.88	0.91	0.64	0.78	0.41
		Mesh size	0.65	0.81	0.16	0.37	0.28
		Sampling time	<0.001 ***	<0.001 ***	<0.001 ***	<0.001 ***	<0.001 ***
2018	Gongzhuling	Maize variety	0.64	0.85	0.56	0.58	0.86
		Mesh size	0.11	0.91	0.86	0.16	0.75
		Sampling time	0.06	<0.001 ***	<0.001 ***	<0.001 ***	<0.001 ***

‘**’ indicates significance at *p* ≤ 0.01; ‘***’ indicates significance at *p* ≤ 0.001.

**Table 2:** Effects of maize variety, mesh size, and sampling time on litter decomposition rate (three-way ANOVA, 2016–2018).

Region	Factor	*p* Value		
		2016	2017	2018
Gongzhuling	Maize variety	0.80	0.16	0.79
	Mesh size	<0.001 ***	<0.001 ***	<0.001 ***
	Sampling time	<0.001 ***	<0.001 ***	<0.001 ***
Shangzhuang	Maize variety	0.63	0.25	—
	Mesh size	<0.001 ***	<0.001 ***	—
	Sampling time	<0.001 ***	<0.001 ***	—
Xinxiang	Maize variety	0.19	0.08	—
	Mesh size	<0.001 ***	<0.001 ***	—
	Sampling time	<0.001 ***	<0.001 ***	—

‘***’ indicates significance at *p* ≤ 0.001; ‘—’, not determined.

**Table 3:** Significant differences in litter decomposition rate among mesh sizes (LSD test, 2016–2018).

Year	Region	*p* Value		
		Mesh Size	Medium	Small
2016	Gongzhuling	Large	0.25	<0.001 ***
		Medium	—	<0.001 ***
	Shangzhuang	Large	0.51	<0.001 ***
		Medium	—	<0.001 ***
	Xinxiang	Large	0.62	<0.001***
		Medium	—	<0.001 ***
2017	Gongzhuling	Large	0.36	<0.001 ***
		Medium	—	<0.001 ***
	Shangzhuang	Large	0.92	<0.001 ***
		Medium	—	<0.001 ***
	Xinxiang	Large	0.07	<0.001 ***
		Medium	—	<0.001 ***
2018	Gongzhuling	Large	0.30	<0.001 ***
		Medium	—	<0.001 ***

‘***’ indicates significance at *p* ≤ 0.001; ‘—’, not determined.

**Table 4:** Effects of maize variety, mesh size and sampling time on the indices of fungal and bacterial (three-way ANOVA).

Microbial Groups	Factor	*p* Value			
		Species Richness	Simpson’s Diversity Index	Shannon–Wiener Index	Pielou’s Evenness Index
Fungal	Maize variety	0.51	0.38	0.62	0.62
	Mesh size	0.47	0.60	0.26	0.31
	Sampling time	<0.001 ***	<0.001 ***	<0.001 ***	<0.001 ***
Bacterial	Maize variety	0.30	0.06	0.35	0.39
	Mesh size	0.94	0.93	0.95	0.96
	Sampling time	<0.001 ***	<0.001 ***	<0.001 ***	<0.001 ***

‘***’ indicates significance at *p* ≤ 0.001.

**Table 5:** Correlation between the decomposition rate of litter and the abundance of soil fauna, the diversity indices of fungal and bacterial.

Variables	Decomposition Rate of Litter	
	Correlation Coefficient (r)	*p* Value
Maize variety	0.015	0.92
Soil fauna abundance	0.847	<0.001 ***
Fungal abundance	0.144	0.30
Fungal Shannon–Wiener index	−0.086	0.54
Fungal Simpson’s index	0.002	0.99
Fungal species richness	−0.058	0.68
Fungal Pielou’s evenness index	−0.086	0.54
Bacterial abundance	−0.013	0.93
Bacterial Shannon–Wiener index	0.082	0.56
Bacterial Simpson’s index	0.055	0.69
Bacterial species richness	0.048	0.73
Bacterial Pielou’s evenness index	0.065	0.65

‘***’ indicates significance at *p* ≤ 0.001.

## Data Availability

The raw data supporting the conclusions of this article are available from the corresponding author upon reasonable request.

## Supplementary Materials

The following supporting information can be downloaded at: https://www.mdpi.com/article/10.3390/ani16193064/s1, Table S1. Effects of maize variety, mesh size, and sampling time on the abundance of dominant fungal OTUs (three way ANOVA). Table S2. Effects of maize variety, mesh size, and sampling time on the abundance of dominant bacterial OTUs (three way ANOVA).
