# Supplementary material for: Soil Fauna Accelerate Litter Decomposition of Cry1Ac Transgenic Maize Independently of Microbial α-Diversity: A Multi-Year, Multi-Region Field Study
Source: Animals (Basel). 2026 Sep 29;16(19):3064. doi: 10.3390/ani16193064 (PMC13663423; doi:10.3390/ani16193064)
Supplement: Supplementary file 1 [file animals-16-03064-s001.zip › animals-4582646-supplementary.pdf]

Table S1. Effects of maize variety, mesh size, and sampling time on the abundance of dominant fungal OTUs (three-way ANOVA).

OTU group	Maize variety		Mesh size		Sampling time	
	F	P	F	P	F	P
OTU1	0.54	0.47	0.23	0.80	105.82	< 0.001***
OTU2	0.13	0.72	2.09	0.14	66.47	< 0.001***
OTU3	2.41	0.13	1.27	0.29	77.33	< 0.001***
OTU4	2.81	0.10	0.33	0.72	75.63	< 0.001***
OTU5	2.41	0.13	1.28	0.29	133.31	< 0.001***
OTU6	0.03	0.85	1.51	0.23	12.70	< 0.001***
OTU7	0.01	0.93	0.46	0.64	9.82	< 0.001***
OTU8	1.87	0.18	2.72	0.08	8.76	< 0.001***
OTU92	0.82	0.37	1.29	0.29	141.11	< 0.001***
OTU9	0.02	0.89	2.40	0.11	132.13	< 0.001***
OTU16	0.11	0.74	1.39	0.26	58.26	< 0.001***
OTU10	0.14	0.71	3.98	0.02*	29.77	< 0.001***
OTU11	0.23	0.63	0.50	0.61	17.33	< 0.001***
OTU13	0.06	0.81	2.24	0.12	39.13	< 0.001***
OTU12	0.16	0.69	0.99	0.38	61.68	< 0.001***
OTU14	0.30	0.59	0.02	0.98	21.31	< 0.001***

'*' indicates significance at P ⩽ 0.05; '**' indicates significance at P ⩽ 0.01; '***' indicates significance at P ⩽ 0.001; OTU, operational taxonomic unit.

Table S2. Effects of maize variety, mesh size, and sampling time on the abundance of dominant bacterial OTUs (three-way ANOVA).

OTU group	Maize variety		Mesh size		Sampling time	
	F	P	F	P	F	P
OTU1	1.24	0.27	1.12	0.34	138.75	< 0.001***
OTU2	0.65	0.43	0.10	0.91	97.25	< 0.001***
OTU7	0.95	0.34	3.22	0.06	15.78	< 0.001***
OTU96	0.00	0.99	3.23	0.06	27.15	< 0.001***
OTU15	0.16	0.69	1.96	0.16	6.62	< 0.004**
OTU9	1.18	0.29	0.66	0.53	20.03	< 0.001***
OTU8	0.63	0.43	1.03	0.37	2.30	0.12
OTU11	3.42	0.07	0.11	0.89	27.34	< 0.001***
OTU5	0.31	0.58	0.46	0.64	7.33	< 0.002**
OTU4	0.04	0.85	0.59	0.56	27.27	< 0.001***
OTU3	0.55	0.46	0.89	0.42	34.83	< 0.001***
OTU10	4.23	0.04*	2.38	0.11	31.14	< 0.001***
OTU6	0.94	0.34	3.74	0.03*	6.32	< 0.004**
OTU18	0.01	0.91	2.61	0.09	91.45	< 0.001***
OTU256	0.50	0.49	1.01	0.37	28.91	< 0.001***
OTU12	2.49	0.12	2.98	0.06	3.69	0.04*

‘*’ indicates significance at $P \leq 0.05$; ‘**’ indicates significance at $P \leq 0.01$; ‘***’ indicates significance at $P \leq 0.001$; OTU, operational taxonomic unit.
